# Expression of Genes Involved in Banana (*Musa* spp.) Response to Black Sigatoka

**DOI:** 10.3390/cimb46120837

**Published:** 2024-12-11

**Authors:** Sávio Luiz Pereira Nunes, Julianna Matos da Silva Soares, Anelita de Jesus Rocha, Fernanda dos Santos Nascimento, Andresa Priscila de Souza Ramos, Taliane Leila Soares, Rogério Merces Ferreira Santos, Vanusia Batista de Oliveira Amorim, Edson Perito Amorim, Claudia Fortes Ferreira

**Affiliations:** 1 Department of Plant Genetic Resources, Federal University of the Reconcavo of Bahia, Cruz das Almas 44380-000, Bahia, Brazil; savionunes12@gmail.com; 2 Department of Biological Sciences, Feira de Santana State University, Feira de Santana 44036-900, Bahia, Brazil; juliannamatos91@gmail.com (J.M.d.S.S.); anelitarocha@gmail.com (A.d.J.R.); feel.20@hotmail.com (F.d.S.N.); talialeila@gmail.com (T.L.S.); rmfsantos@uefs.br (R.M.F.S.); 3 Embrapa Mandioca e Fruticultura, Cruz das Almas 44380-000, Bahia, Brazil; andresa.ramos@embrapa.br (A.P.d.S.R.); vanusiaamorim50@gmail.com (V.B.d.O.A.); edson.amorim@embrapa.br (E.P.A.)

**Keywords:** gene expression, *Musa* spp., *Mycosphaerella fijiensis*, RT-qPCR

## Abstract

This work aimed to evaluate the relative gene expression of the candidate genes *psI*, *psII*, *isr*, *utp*, and *prk* involved in the defense response to Black Sigatoka in banana cultivars Calcutta-4, Krasan Saichon, Grand Nain, and Akondro Mainty, by a quantitative real-time PCR. Biotic stress was imposed on 6-month-old plants during five sampling intervals under greenhouse conditions. The *psII* and *isr* genes were upregulated for the Calcutta-4- and Krasan Saichon-resistant cultivars, and were validated in this study. For Grande Naine, a susceptible cultivar, there was an early downregulation of the *psI*, *psII*, and *isr* genes and a late upregulation of the *psII* gene. There was no significant expression of any of the genes for the susceptible cultivar Akondro Mainty. Computational biology tools such as ORFFinder and PlantCARE revealed that the utp gene has more introns and exons and that, in general, cis-elements involved in the response to biotic stress, such as as-1, w-box, and STRE, were detected in the promoter region of the genes studied. Data from this work also support the phenotyping studies of banana cultivars affected by Black Sigatoka in the field. Once validated in promising new hybrids, these genes may be used in marker-assisted selection (MAS) and/or gene-editing techniques.

## 1. Introduction

Bananas feed more than 500 million people and are grown mainly in tropical and subtropical regions worldwide where they play a key role in food security [1]. In 2022, the world production of bananas was approximately 115.7 million tons with a harvested area of around 5.7 million hectares [1]. The largest banana producers in the world are India, China, Indonesia, and Brazil.

However, the low level of technology adopted and the problems caused by biotic and abiotic stresses have drastically affected the banana production system. Fusarium wilt (*Fusarium oxysporum* f. sp. *cubense*) and Yellow (*Mycosphaerella musicola*) and Black (*Mycosphaerella fijiensis*) Sigatokas are the main fungal diseases affecting banana production [2]. Among the sigatokas, black leaf streak disease (BLSD) is the most devastating.

The etiological agent of Black Sigatoka is the hemibiotrophic fungus *Mycosphaerella fijiensis Morelet* [anamorphic phase *Pseudocercospora fijiensis* (*Morelet*) *Deighton*] belonging to the phylum Ascomycota. The cycle of *M. fijiensis* is composed of the germination phases of asexual (conidia) and/or sexual (ascospores) spores, followed by the penetration into the host, the development of symptoms, and, finally, the production of conidia and/or ascospores [3]. The main symptoms of the disease are characterized by very small spots that coalesce into black streaks parallel to the leaf venation, which fuse, causing necrosis.

The leaf spots reduce the photosynthetic area, leading to slow plant growth and development, early fruit maturation, and low productivity and quality of fruits [4]. Chemical control is currently the most used method and is carried out by applying systemic and protective fungicides, which implies high costs for producers. Therefore, the most environmentally friendly means of control is the use of resistant varieties [5].

Embrapa is one of the five institutions worldwide that carries out a banana genetic breeding program, with one of the largest *Musa* spp. and plantain collections in the world. The cultivars used in our work are contrasting for resistance to Black Sigatoka, namely, Calcutta-4 and Krasan Saichon, resistant to BLSD, and Akondro Mainty and Grande Naine, susceptible to BLSD. These cultivars are important to the banana genetic breeding program (BGBP) carried out at Embrapa, aiming to obtain banana cultivars resistant to Black Sigatoka, and have been thoroughly evaluated at the field level for phenotypic characteristics concerning the disease [6].

Previous studies identified genes involved in the defense response to Black Sigatoka in banana plants by transcriptome and gene expression analysis [4,7,8,9,10]. The genes involved in the banana defense response to Black Sigatoka include *psI*, *psII*, *isr*, *utp*, and *prk*, previously identified in the *Musa* spp. × *M. fijiensis* interaction by Timm et al. [9] and Mendoza-Rodríguez et al. [10], and were selected for the present study.

The *psI* and *psII* genes encode the N-subunit of the Photosystem I and Photosystem II reaction center, and are related to the primary metabolism. The expression of the *psI* and *psII* genes are key since they are involved in the synthesis of immune response mediators, such as reactive oxygen species (ROS) and hormones [11].

The *isr* gene encodes the isoflavone reductase, an enzyme involved in the biosynthesis chain of medicarpine which plays an important role in the phenylpropanoid- or flavonoid-derived pathway, one of the pathways responsible for the production of secondary plant compounds, such as isoflavones, lignins, and anthocyanins [12].

In addition to this gene, the *utp* gene also acts in defense against Black Sigatoka. This gene encodes glucose-1-phosphate uridylyltransferase, an enzyme with a key role in the metabolism of galactose, starch, and sucrose, involved in the synthesis of the plant cell wall, one of the main barriers to infection by pathogens [13].

Finally, the *prk* gene, a phosphoribulokinase encoder, has several functions, such as kinase and phosphotransferase activity. The phosphoribulokinase enzyme plays an important role in carbon fixation, as it generates the C5 carbon substrate, essential for Rubisco activity (ribulose 1,5-bisphosphate carboxylase/oxygenase) [14,15].

Therefore, the present study aims to validate candidate genes for resistance to Black Sigatoka through gene expression analysis, in addition to detailing these genes of interest and the proteins encoded by them through bioinformatics tools, which can be used to analyze the expression and regulation of genes and proteins, compare genetic and genomic data, and understand the evolutionary aspects of biology at the molecular level, among other applications. This validation is important for further studies related to MAS and gene editing via CRISPR-Cas9 in *Musa* spp.

## 2. Materials and Methods

### 2.1. Genetic Material

**Figure 1:**
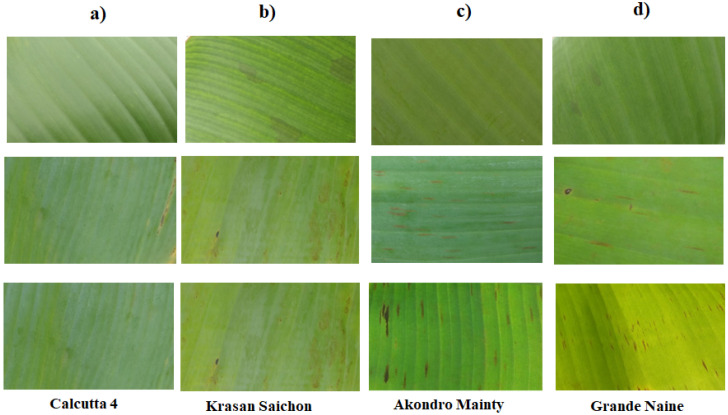
Progress of Black Sigatoka symptoms in contrasting banana genotypes from the Embrapa Mandioca e Fruticultura Germplasm Bank, evaluated by Nascimento et al. [6]: (**a**) evaluation carried out after candle leaf issuance; (**b**) 8 days after the first evaluation; (**c**) 15 days after the first evaluation; and (**d**) 30 days after the first evaluation. Photo: Nascimento, 2021 [6].

The genetic material used in this work consisted of four contrasting banana cultivars as to resistance to Black Sigatoka (Table 1) originating from the germplasm collection at Embrapa Mandioca e Fruticultura, located in Cruz das Almas, Bahia, Brazil. The diploid Calcutta-4 and triploid Grande Naine cultivars are described in the literature as resistant (R) and susceptible (S) to the disease, respectively [16,17]. In contrast, the diploids Krasan Saichon and Akondro Mainty were chosen from previous field phenotyping experiments where they showed resistance and susceptibility to BLSD, respectively [6]. The accessions evaluated by Nascimento et al. [6] were classified as resistant and susceptible according to the scale of grades proposed by Stover (1972) and later modified by Gauhl (1989): (1) up to 1%; (2) 1 to 5%; (3) 6 to 15%; (4) 16 to 33%; (5) 34 to 50%; and (6) 51 to 100% of the leaf area damaged. It is possible to see the progression of the disease symptoms in the contrasting cultivars in Figure 1.

Furthermore, Krasan Saichon and Akondro Mainty are close ancestors of commercial materials and possible ancestors of the subgroup of AAA cultivars known as Cavendish.

Therefore, the three diploids and the Grande Naine cultivar were chosen, given their importance to the banana genetic breeding program, aiming to develop BSLD-resistant cultivars and with appealing organoleptic characteristics, among others.

### 2.2. Bioassay: Musa spp. x Mycosphaerella Fijiensis Pathosystem

The bioassay was carried out in the greenhouse at Embrapa Mandioca e Fruticultura located in Cruz das Almas, Bahia, Brazil, with geographic coordinates 12°40′19″ S and 39°06′22′ W, 220 m above sea level. The average annual temperature is 24.5 °C, and the average annual rainfall is 1250 mm [18]. The experimental design was completely randomized, with three replicates.

The isolation of *M. fijiensis* was carried out through the indirect method (wet chamber), from infected leaves of the Grand Nain cultivar from the germplasm collection at Embrapa Mandioca e Fruticultura. Leaf fragments in stages 4 to 6 of disease development were washed in running water and neutral detergent for mycelium growth, placed in Gerbox (acrylic box with lid) containers with moist cotton, and stored in BOD at 25 °C with a 12 h photoperiod. Afterwards, the leaf fragments were transferred to Petri dishes filled with PDA medium to obtain a pure culture.

The inoculum used consisted of a 10 mL suspension of 4 × 10^4^ mL^−1^ of *M. fijiensis* mycelium fragments. At 6 months, leaves 1 and 2, which represent the first and second youngest leaves, expanded, respectively, were inoculated in a greenhouse (with controlled temperature (25 °C) and relative humidity maintained between 85 and 90%), where the suspension was brushed on the adaxial part of the leaves, a method suggested by Leiva-Mora et al. [19]. The brushed leaves were wrapped in a plastic bag and cotton moistened with distilled water to create an appropriate environment for a successful fungal infection.

Fifteen plants per cultivar, totaling sixty plants (samples), were used in the experiment with the following time points for collection: 0 (control—control plants), 3, 9, 15, and 21 days after inoculation (DAI). These time points were chosen because the genes to be validated respond in the earlier stages of the disease [7,9,10].

At each established time point, the middle third of leaf 3 of 3 plants per cultivar was collected with alcohol-sterilized scissors. Afterwards, the samples were wrapped in aluminum foil and immediately frozen in liquid nitrogen and stored in an ultrafreezer at −80 °C until further analysis.

### 2.3. Molecular Analysis

#### 2.3.1. RNA Extraction and Treatments

Total foliar RNA was extracted from 100 mg of tissue from each plant following a modified version of the protocol described by Zhao et al. [20]. The integrity of the extracted RNA was verified by electrophoresis on a 1.0% agarose gel stained with ethidium bromide. The RNA was subjected to DNase treatment using the TURBO DNA-free DNase Treatment kit (Ambion, Austin, TX, USA) following the protocol established by the manufacturer and stored in an ultrafreezer (−80 °C).

#### 2.3.2. cDNA Synthesis and Viability

The cDNA synthesis was performed using the High-Capacity RNA-to-cDNA kit (Applied Biosystems, Foster City, CA, USA) following the manufacturer’s specifications. Afterwards, the cDNA samples were quantified in a Nanodrop ND-2000 (Thermo Scientific, Waltham, MA, USA) spectrophotometer, diluted to 100 ng/µL, and stored in ultrafreezer −80 °C.

The verification of cDNA viability consisted of a conventional PCR using the 25S primer (reference gene for *Musa* spp.) [21]. The PCR mix with a final volume of 20 µL consisted of 3 µL of buffer 5×, 1.6 µL of magnesium chloride at 25 mM, 1.2 µL of 2.5 mM dNTP, 3 µL of primers (forward and reverse primers) at 3 µM, 0.2 µL of Taq DNA Polymerase (Promega, Madison, WI, USA), and 5 µL of cDNA. PCR products were quantified on a 2% agarose gel and stained with ethidium bromide.

#### 2.3.3. Primer Design

The gene loci provided by Mendoza-Rodríguez et al. [10] and Timm et al. [9] were submitted to The Banana Genome Hub database (https://banana-genome-hub.southgreen.fr/ accessed on 4 January 2023) [22] in order to obtain the complete genetic sequence involved in the defense response to Black Sigatoka. These sequences were used to design the primers using the Primer Express (Applied Biosystems, Waltham, MA, USA, v. 2.0) software following the parameters required for the quantitative real-time PCR [23].

#### 2.3.4. Validation of Reference Genes

The reference genes used for the gene expression analysis in the banana genotypes in our work were 25S, tubulin, elongation factor 1 (EF1), and actin 1 (GSMUA_Achr6G25350_001) (Table 2).

The primers used were described by Podevin et al. [21], except for actin 1, which was designed in this study. A conventional PCR test was performed, followed by a qPCR to test the amplification efficiency of the primers.

The conventional PCR mix with a final volume of 20 µL consisted of the following: 2 µL of buffer 10×, 1.6 µL of magnesium chloride at 25 mM, 1.2 µL of dNTP at 2.5 mM, 2 µL of primers at 10 µM, 1 µL of Taq DNA Polimerase (Cellco, Austin, TX, USA), and 5 µL of cDNA. PCR products were quantified on a 1.0% agarose gel and stained with ethidium bromide.

Primer efficiency tests were performed using a standard curve and resulting R^2^ in the ABI 7500 Fast Real-Time PCR System (Applied Biosystems) at a final volume of 10 μL. The mix was composed of the following: 1 μL cDNA in serial dilution (100, 50, 25, 12.5 and 6.25 ng/μL), 1 pair of primers (0.3 μL forward and reverse primers at 10 μM), 5 μL of Sybr Green PCR mix (Ludwig Biotech), and 3.5 μL of nuclease-free water.

#### 2.3.5. Gene Expression Analysis

The analysis of the expression of the *psI*, *psII*, *isr*, *utp*, and *prk* genes involved in response to Black Sigatoka in the banana samples was carried out through the qualitative real-time PCR technique in the ABI 7500 Fast Real-Time PCR System (Applied Biosystems). The synthesized cDNA was diluted at 100 ng/μL and used in the reaction with Sybr Green PCR mix (Ludwig Biotech, Alvorada, Brasil) at the following ratios: 3.5 μL of Sybr Green mix, 1 μL of cDNA, and primers at the final concentration of 3 μM (PsI and PsII), and 2.5 μM, in 10 μL final volume.

Technical triplicates of each leaf sample were used for each of the genes analyzed, and biological triplicates were used for each banana cultivar. RT-qPCR took place under the following conditions: 50 °C (20 s), 95 °C (10 min), followed by 40 cycles of denaturation at 95 °C (15 s), and annealing and extension at 57 °C (Isr primer) and 59 °C (PSI primers, PsII, 25S, and ACT1) (60 s). The melting curve was generated from the denaturation of the amplified product at the end of the reaction in order to detect possible contaminants or primer dimer formations (95 °C—15 s, 60 °C—60 s, and 95 °C—15 s) with fluorescence signal detection at the end of each extension step.

The data were evaluated using the relative quantification method of the comparative optimal quantification cycle (Cq) (ΔΔCq). The Cqs were calculated using the Real-Time PCR Miner algorithm [24]. The ratio between mRNA levels of treatment (3, 9, 15, and 21 DAI) and control (0 DAI) plants was statistically calculated using the REST 2009 software version 2.0.13 [25] based on the Pairwise Fixed Reallocation Randomization Test, as described by [26]. This test considers a significant range of *p* < 0.05. For data normalization, the Actin 1 gene, designed in the present study and the 25*S* gene (Table 2), according to [21], were used.

### 2.4. Bioinformatics Analysis

#### 2.4.1. Gene Analysis

In addition to the study of gene expression, analysis was performed using bioinformatics tools in order to provide more information on the validated genes and proteins encoded by them, to enrich the knowledge of such genes within the pathosystem. The gene loci provided by Timm et al. [9] and Mendoza-Rodríguez et al. [10] were submitted to The Banana Genome Hub database (https://banana-genome-hub.southgreen.fr/ (accessed on 28 October 2024)) [22] in order to retrieve the complete sequence of the genes.

The CDS sequence of the genes as well as the transcripts and the amino acid sequences were obtained in the Phytozome Platform (https://phytozome.jgi.doe.gov/pz/portal.html# (accessed on 28 October 2024)) [27]. The Open Reading Frame (*ORF*) 1 was identified with the aid of the *ORFFinder* software (https://www.ncbi.nlm.nih.gov/orffinder/) in the *NCBI* database (https://www.ncbi.nlm.nih.gov/) [28]. The promoter region (1.5 kb) of each gene was scanned in the PlantCARE database (http://bioinformatics.psb.ugent.be/webtools/plantcare/html/ (accessed on 28 October 2024)) [29] to identify cis-action regulators and their functions.

#### 2.4.2. Protein Analysis

Protein analysis was conducted by verifying post-translational modifications. Phosphorylated residues of the Tyrosine (Tyr), Threonine (Thre), and Serine (Ser) amino acids were detected using the NetPhos 3.1 server (http://www.cbs.dtu.dk/services/NetPhos/ (accessed on 28 October 2024)) [30] in all five proteins encoded by the genes involved in response to Black Sigatoka. N-type glycosylation sites were verified using the *NetNGlyc* 1.0 server (http://www.cbs.dtu.dk/services/NetNGlyc/ (accessed on 28 October 2024)) [31].

Through the conserved protein domain, a search was performed in the Pfam 32.0 database (https://www.ebi.ac.uk/interpro/entry/pfam/ (accessed on 28 October 2024) [32] for the families to which the analyzed proteins belong. With the help of the ProtParam tool (https://web.expasy.org/protparam/) of the ExPasy portal (https://web.expasy.org/) [33], physical and chemical information on proteins, namely, molecular weight, isoelectric point, stability index, aliphatic index, and average hydropathicity, were collected.

The subcellular location of proteins was checked by *DeepLoc*-1.0 predictor (http://www.cbs.dtu.dk/services/DeepLoc/ (accessed on 28 October 2024)) [34] and the presence of signaling peptide by *SignalP*-5.0 server (http://www.cbs.dtu.dk/services/SignalP/ (accessed on 28 October 2024)) [35]. Finally, the conserved protein motifs were detected through the *MEME Suite* 5.1.1 software (http://meme-suite.org/tools/meme) [36].

## 3. Results and Discussion

### 3.1. Molecular Analysis

Reference primers EF1 and *β*-tubulin amplified a fragment in the corresponding bp, but presented an unspecific band and were not used in this study. Primers 25S and actin 1 (ACT 1) were tested in a conventional PCR at annealing temperatures of 59 and 60 °C, respectively. Samples referring to 3 DAI of cultivars Calcutta-4 and Krasan Saichon were randomly chosen. It was possible to obtain the expected amplicons for each primer, being 106 bp for 25S and 80 bp for ACT1. Therefore, these two primers were tested for efficiency in qPCR and the standard curves are shown in Supplementary Figure S1A,B.

The amplification efficiency indicates how exponential a reaction is, that is, how much the template is doubled at each cycle. Building a standard curve using serially diluted samples is one of the methods for checking the reaction efficiency, considered acceptable when ranging between 80 and 110%. The slope or the angular coefficient of the straight line around −3.322 ensures a high qPCR efficiency. The linear regression coefficient of determination (R^2^) refers to the overlapping of standard samples on the straight line, being around 0.985 in a reaction considered efficient [23].

Therefore, the efficiency of the qPCR reaction using the 25S and ACT1 primers are acceptable, thus being considered as good reference genes for *Musa* spp. in gene expression analysis, supporting the data presented in Portal et al. [7] and Podevin et al. [21]. Identifying and selecting reference genes are fundamental steps in studies with qPCR in non-model plants, such as bananas. Two to three reference genes are recommended to ensure greater gene expression analysis reliability [23]. Primers developed for gene amplification are shown in Table 3.

Primers corresponding to the *psI*, *psII*, *isr*, and *act1* genes were specific to the target region, as evidenced by the melting curve, which has a well-defined peak only, with no primer dimer formation and no contaminants.

On the other hand, the *utp* gene primers proved to be inefficient in amplifying the cDNA template due to the formation of an isolated peak that did not match its amplicon and to failures in the amplification of the samples as a whole. Furthermore, for the *prk* gene primers, several temperature peaks formed, indicating THE amplification of several target molecule fragments. According to [23], an abnormal melting curve reveals problems in qPCR, such as the target molecule quality, pipetting errors, and formation of primer dimers, directly interfering with its efficiency. In view of the above, the *utp* and *prk* genes were not used in the gene expression analysis of the pathosystem *Musa* spp. x *M. fijiensis*.

**Figure 2:**
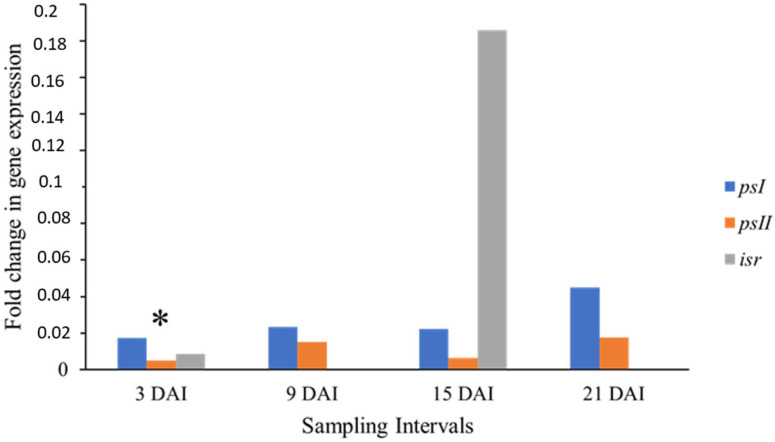
Relative expression of the *psI*, *psII* and *isr* genes between treatments, 3, 9, 15, and 21 DAI, and control (0 DAI) plants of the Calcutta-4 cultivar (resistant to Black Sigatoka) expressed as a ratio of mRNA abundance on a logarithmic scale for each collection interval in days after inoculation (DAI). Data were normalized by actin 1 and 25*S* genes. (*) Expression values with statistically significant difference (*p* < 0.05) by the pairwise fixed-allocation randomization test in the 2009 REST program [25].

In the present study, the expression of three genes was quantified: *psI* (subunit N of Photosystem I reaction center), *psII* (subunit R of Photosystem II), and *isr* (isoflavone reductase). The relative expression of the mRNA levels of these genes in treatments 3, 9, 15, and 21 DAI, and control (0 DAI) for the cultivar Calcutta-4, used as the Black Sigatoka-resistant control, are shown in Figure 2. The psII gene showed an overexpression (upregulation) at 3 DAI, with statistical significance. In turn, the psI gene showed low expression levels at 3, 9, 15, and 21 DAI, without statistical significance. The isr gene showed a peak of expression at 15 DAI, without statistical significance.

This scenario differs from that presented by Mendoza-Rodríguez et al. [10]. They noticed a downregulation of the *psII* gene, and an upregulation of the *psI* gene in Calcutta-4, during an incompatible interaction (resistance) with *M. fijiensis.* In spite of that, those two genes play a fundamental role in the same biological process, photosynthesis. Thus, despite the disparity between the data discussed here and those in the literature, the plant defense strategy, in both cases, is similar.

It is worth mentioning that the baseline defense of plants is initiated with the perception of molecular patterns associated with pathogens (PAMPs), such as flagellin, by pathogen recognition receptors (PRRs), such as leucine-rich kinases. This plant response is named PAMP-triggered immunity (PTI). Therefore, the pathogen produces effectors, which consist of avirulence proteins (Avr) capable of evading PRR recognition, thus causing the so-called effector-activated susceptibility (ETS) in the host. Through an evolutionary process, plants have produced resistance proteins (R), which can recognize, directly or indirectly, avirulence proteins. Thus, a stronger defense response is induced in reaction to ETS, called effector-triggered immunity (ETI), which is associated with the hypersensitive response (HR). These events make up the zig-zag model proposed by [37] to elucidate the early plant defense response and the pathogenic counterattack.

Furthermore, the resistance observed in Calcutta-4 is obtained through HR, as characterized by the apoptosis of plant cells at the site of infection due to toxin release. These destroy the fungus and prevent its systemic colonization and, therefore, the development of the disease, as observed by Churchill [3], Timm et al. [9], and Arango Isaza et al. [38].

Moreover, the chloroplast is one of the key organelles in generating defense mechanisms against phytopathogens, such as the synthesis of reactive oxygen species (ROS) and hormones [39]. The energy required for the plant defense comes from the photosynthetic activity, but this capacity is compromised since the infection by *M. fijiensis* causes leaf tissue necrosis [40].

Therefore, a fundamental characteristic for a Black Sigatoka-resistant cultivar, in addition to presenting HR, would be to overcome this drop in photosynthetic capacity by the overexpression of genes related to the photosynthesis machinery, as is the case of the *psII* gene, which contributes to the dissipation of light energy in the PAMP recognition stage [41]. The early Calcutta-4 upregulation may support its disease-resistant character.

On the other hand, the *isr* gene did not show statistical significance in the two intervals in which its expression was detected, at 3 and 15 DAI. Mendoza-Rodríguez et al. [10] reported a mild induction of this gene expression early in the infection, followed by a repression after the 9 DAI time point, supporting this work’s data.

**Figure 3:**
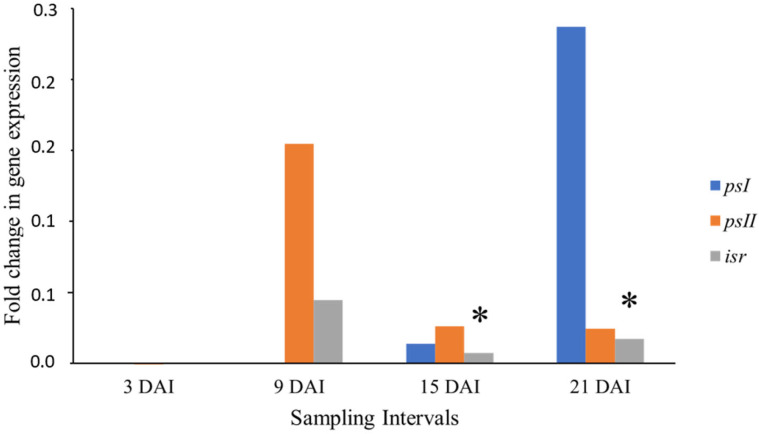
Relative expression of the *psI*, *psII*, and *isr* genes between treatments 3, 9, 15, and 21 DAI and control (0 DAI) plants of the Krasan Saichon cultivar (resistant to Black Sigatoka) expressed as a ratio of mRNA abundance on a logarithmic scale for each collection interval in days after inoculation (DAI). Data were normalized for actin 1 and 25*S* genes. (*) Expression values with statistically significant difference (*p* < 0.05) by the pairwise fixed-allocation randomization test in the 2009 REST program [25].

The relative gene expression given by fold changes of the ratio of mRNA levels between the treatment and control plants of the Krasan Saichon cultivar (Black Sigatoka-resistant) is shown in Figure 3.

At 3 DAI, there was almost no expression of the genes analyzed. The *psI* gene underwent induction at 15 DAI, followed by repression at 21 DAI. The *psII* gene expression underwent a slight induction at 9 DAI. There was no statistically significant expression for these two genes. Regardless, the expression of these genes may indicate that Krasan Saichon shows a late response to the disease compared to Calcutta-4, the resistant control cultivar. Nonetheless, the number of necrotic lesions would not increase to the point of indicating cultivar susceptibility since the fungus takes 3 to 4 weeks to reach the necrotrophic phase [3].

The *isr* gene was upregulated with a statistically significant expression at 15 and 21 DAI (Figure 3). This gene plays an important role in isoflavonoid biosynthesis (via phenylpropanoid). This route is responsible for producing phenolic compounds that act on the lignification, cell wall fortification, and phytoalexins synthesis in plant defense against *M. fijiensis* [42].

Phytoalexins are low-weight antimicrobial compounds synthesized by plants during abiotic and biotic stress, which play an important role in the defense against pathogens. Pisatin and phaseolin are examples of phytoalexins. The former is produced in *Pisum sativum* (pea) and the latter in *Phaseolus vulgaris* (beans). In both cases, they accumulate at the infection site in fungitoxic concentrations, which further supports the action of these secondary metabolites in plant defense [43,44,45].

Mendoza-Rodríguez et al. [10] reported the upregulation of the *isr* gene in the incompatible interaction, Calcutta-4 x *M. fijiensis*, and downregulation in the compatible (susceptible) Grand Nain x *M. fijiensis* interaction. In the latter, downregulation coincided with the onset of the disease symptoms.

Krasan Saichon is a cultivar from the Banana Germplasm Collection at Embrapa, characterized as Black Sigatoka-resistant in a previous phenotyping study carried out in the Recôncavo region [6]. The literature still does not address the disease-response gene expression in this cultivar; however, the significant expression of the *isr* gene contributes to the understanding of the phenylpropanoid pathway’s fundamental relevance in defense of *Musa* spp., as reported by [7], and further supports the resistant character of this cultivar as observed in the field. However, further studies are needed to elucidate the molecular mechanism of the response to Black Sigatoka, exhibited by Krasan Saichon.

**Figure 4:**
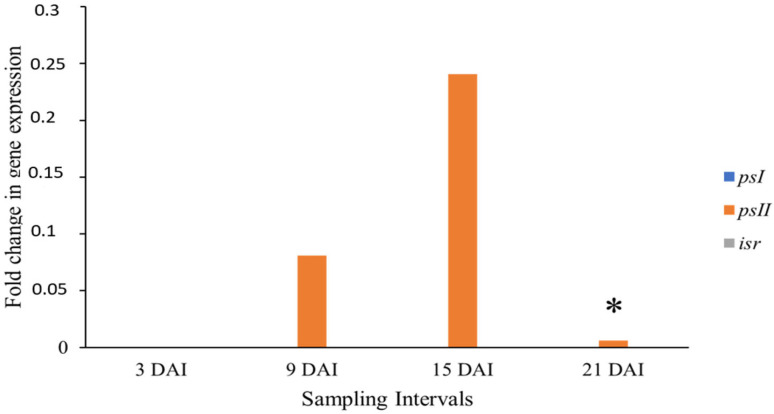
The relative expression of the *psII* gene between treatment (3, 9, 15, and 21 DAI) and control (0 DAI) plants of the Grand Naine cultivar (susceptible to Black Sigatoka) expressed as a ratio of mRNA abundance on a logarithmic scale for each collection interval in days after inoculation (DAI). Data were normalized for actin 1 and 25*S* genes. (*) Expression values with statistically significant difference (*p* < 0.05) by the pairwise fixed-allocation randomization test in the 2009 REST program [25].

Figure 4 shows the relative expression between the treatment and control plants of the cultivar Grand Nain (Susceptible to BLSD). There was a statistically significant downregulation of the three genes at 3 DAI. The *psII* gene was the only one expressed in the following intervals. At 9 and 15 DAI its expression was low, without statistical significance; however, at 21 DAI, this gene was upregulated, with statistical significance.

Mendoza-Rodríguez et al. [10] reported the complete repression of the *psI* and *psII* genes in *M. fijiensis*-infected Grand Nain. As the Grand Nain susceptibility to BLSD is widely discussed and confirmed in the literature, this cultivar was used as the susceptible control in the present study. The late upregulation of the *psII* gene may characterize the plant’s response to pathogen invasion and contribute to its susceptible character, as this gene helps dissipate excess light energy in the PAMP recognition stage [41]. This late regulation directly contrasts with the early upregulation of the *psII* gene in Calcutta-4, as verified in this work and which contributes to disease resistance. Therefore, it can be inferred that the early expression of the *psII* gene is related to the resistance to Black Sigatoka.

It is also worth noting that studies have identified phytoanticipins in Grand Nain cultivars challenged by *M. fijiensis*. These low-molecular-weight antimicrobial compounds are present in plant tissue at baseline, in addition to being produced in larger quantities from pre-existing molecules upon pathogen infection. Despite the presence of these secondary metabolites across the coevolution process of the *M. fijiensis* x Grand Nain pathosystem, the pathogen has shown greater success, probably due to its ability to evade host defenses through avirulence proteins. Understanding the action of phytoanticipins in plant defense can contribute to the induction of resistance mechanisms in susceptible banana cultivars [3,46].

**Figure 5:**
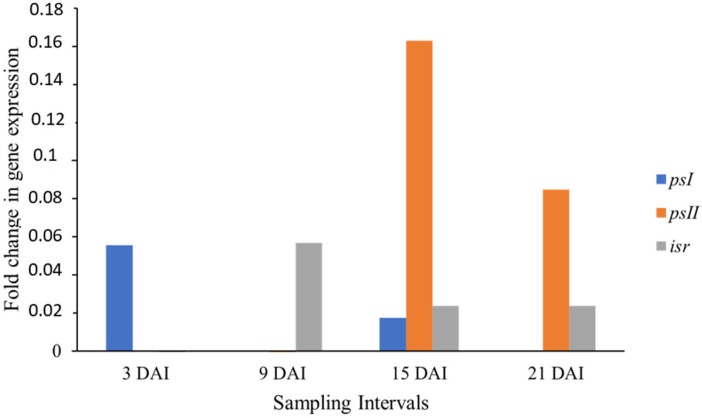
The relative expression of the *psI*, *psII*, and *isr* genes between treatment 3, 9, 15, and 21 DAI and control (0 DAI) plants of the cultivar Akondro Mainty (susceptible to BLSD) expressed as a ratio of mRNA abundance on a logarithmic scale for each collection interval in days after inoculation (DAI). Data were normalized for actin 1 and 25*S* genes.

The relative expression between treatment and control plants of the Akondro Mainty cultivar (Susceptible to BLSD) is shown in Figure 5.

The *psI* gene was expressed at 3 and 15 DAI, when it reached its peak. The *psII* gene was expressed late and at low levels at 15 and 21 DAI. In contrast, the *isr* gene was expressed at 9, 15, and 21 DAI. None of the genes showed a statistically significant expression. Regardless, the late expression of the *isr* and *psII* genes, and the irregular expression of the *psI* gene, may indicate the susceptible character of the cultivar observed in a previous field phenotyping study [6]. Similar to Krasan Saichon, Akondro Mainty comes from Germplasm Bank of Embrapa, and there are still no studies addressing the gene expression in response to the BLSD resistance for these diploids.

### 3.2. Bioinformatics Analysis

In order to thoroughly analyze the candidate genes involved in the defense response to Black Sigatoka, a computational biology analysis was performed. By using bioinformatics tools, the intronic and exonic sequences, and the open reading and promoter regions of the genes, were analyzed, as well as the post-translational modifications and the motifs of the proteins encoded by them.

The analysis of the five genes involved in the defense response to BLSD evidenced the number of intronic and exonic sequences of each, as shown in Table 4.

The *psI*, *psII*, *isr*, and *prk* genes have shorter sequences, with a lower number of introns and exons when compared to the *utp* gene. The more introns a gene has, the longer it takes to process the mRNA particularly during the splicing mechanism. Introns represent a large part of the genome; they are larger in base pairs when compared to exons; and they conserve important nucleotides and have been maintained by natural selection [47]. Intronic sequences also play an important role during alternative splicing, consisting of generating different transcripts from a single gene, and in gene expression, acting as regulators, the so-called microRNAs (miRNAs) [48].

Furthermore, the open reading frame (ORF) of a gene corresponds to the sequence that starts at a start codon (ATG) and ends at one of the three stop codons (TAG, TGA, and TAA). Detecting an *ORF* is a key step in searching for protein encoder genes [49] since gene reading is the starting point. The size of the open reading frame of the genes studied, and the number of amino acids of the proteins encoded by each of them, is shown in Table 5.

*Cis* elements are transcriptional regulatory units present in a gene’s promoter region (5′ UTR untranslated region). Each gene’s unique combination of these elements determines its spatial and temporal expression. Many biological processes and responses to biotic and abiotic stress are controlled by cis elements [50]. Therefore, the analysis of the gene promoter region revealed several cis-action regulatory elements related to hormonal regulation, stress response, and primary metabolic processes [29], in addition to the typical signals involved with the start of transcription, such as the TATA-box and CAAT-box. This suggests a complex regulation of the expression of genes involved in the defense response of bananas to Black Sigatoka.

It is also worth mentioning that the presence of *cis* elements involved in the defense response to fungal elicitors (compounds that stimulate plant defense), such as S-box, CCGTCC-box, and w-box, identified here only in the primary metabolism (Photosystem II) gene *psII*, suggests the important role of this gene in the early response to infection by *M. fijiensis*, since Black Sigatoka is characterized by a reduction in the plant’s photosynthetic capacity [51].

Finally, Table 6 shows the most frequent *cis* elements related to biotic stress in the promoter region of the studied genes. The activation sequence-1 (as-1) and w-box elements are induced by the presence of a pathogen, while the stress-response element (STRE) responds to stress in general. Redman et al. [52] observed that biotic stress differentially stimulated the activity of the as-1 element in *Arabidopsis thaliana*, while Choudhury et al. [53] detected the differential activation of cis elements of genes involved in carbohydrate metabolism due to abiotic stress in bananas.

These studies reinforce the importance of identifying cis elements and their functions in different environmental and physiological conditions of the plant. In this work, the scanning of functional cis elements provides a better understanding of the spatial and temporal expression of genes involved in the plant defense response to Black Sigatoka [51,54].

The compilation of post-translational modifications verified in the proteins involved in the defense response to Black Sigatoka are presented in Table 6, and this presents the general characteristics of the protein molecules investigated in our work. From identifying the functional domain, it was possible to detect the protein families to which the amino acid sequences belong. The first of these families was PSAN or PSI-N (pfam05479 domain), which harbors several proteins from the Photosystem I reaction center, such as the GSMUA_Achr6T25780_001 protein, which is located in the thylakoid lumen and probably mediates the interaction between plastocyanin and the Photosystem I reaction center (Table 7). Studies have shown that this interaction is compromised in *Arabidopsis thaliana* mutants in which the protein encoded by the *psI* gene is absent [55].

The GSMUA_Achr9T30450_001 protein encoded by the *psII* gene belongs to the PsbR family (pfam04725) domain. The literature highlights that this polypeptide is associated with the oxygen evolution complex of Photosystem II and addresses its 10 kDa hydrophobic C-terminal region as a structure that enables the transport of the protein across the thylakoid membrane [56].

Furthermore, the NmrA family (pfam05368) comprises small-chain dehydrogenases and reductases which contribute to the post-translational modification of the transcription factor AreA, involved in nitrogen metabolism. The GSMUA_Achr2T14320_001 (isoflavone reductase) protein, a product of the *isr* gene, is part of this family [57].

The GSMUA_Achr9T23930_001 protein encoded by the *utp* gene is part of the UDPGP family (pfam01704), which houses the glucose-1-phosphate uridylyltransferases. This protein plays an important role in carbohydrate metabolism (glycolysis/gluconeogenesis pathway), as it catalyzes the interconversion that generates UDP-glucose [58].

Clendennen and May [59] reported the differential expression of genes that encode proteins involved in carbohydrate metabolism associated with the response to biotic and abiotic stresses and the ripening of banana fruits. Keller and Ludlow [60] detected the increased activity of amylase and sucrose phosphate synthetase enzymes during water stress in *Cajanus cajan* (pigeon pea) leaves, indicating that carbohydrate metabolism may be involved in increased stress tolerance. The role of proteins involved in carbohydrate metabolism during a water deficit in banana plants was also observed by Muthusamy et al. [61].

Carbohydrate metabolism is also affected in other plant x pathogen interactions, such as *Triticum* spp. x *Puccinia triticina*, causing wheat leaf rust [62]. The glycolysis/gluconeogenesis pathway contains enzymes necessary for other pathways, such as the pathway of the xenobiotic metabolism by cytochrome P450, responsible for processing the fungal compounds after infection and involved in the synthesis of compounds via the phenylpropanoid pathway [63]. These aspects are relevant to this work, as the activation of these pathways by the GSMUA_Achr9T23930_001 protein can generate a more effective defense response to Black Sigatoka right after the penetration of *M. fijiensis* through the stomata, thus supporting the results obtained by Timm et al. [9].

The PRK family (pfam00485 domain) comprises three types of kinases, namely, uridine kinases, bacterial pantotheate kinases, and phosphoribulokinases, such as the GSMUA_Achr5T03660_001 protein, encoded by the *prk* gene. This enzyme converts ribulose 5-phosphate to ribulose 1,5-biphosphate, which is the substrate of the carbon-fixing enzyme Rubisco (ribulose 1,5-biphosphate carboxylase/oxygenase), essential to the biochemical phase of photosynthesis [64].

Al-Obaidi et al. [65] identified high levels of the protein encoded by the *prk* gene during biotic and abiotic stress. In *Nicotiana benthamiana* (tobacco) infected by the pepper mild mottle tobamovirus, activity of this protein was also detected [66], as well as in *Brassica napus* (canola) challenged by *Sclerotinia sclerotiorum* [67]. The identification of the GSMUA_Achr5T03660_001 protein in response to Black Sigatoka supports these results, as well as those of Timm et al. [9], and may indicate the complementation of the photosynthetic capacity compromised in the face of a fungal attack, which ensures the banana crop productivity.

Several cell-signaling processes are influenced by phosphorylation on protein tyrosine, threonine, and serine residues. All characterized proteins showed phosphorylation sites. The GSMUA_Achr6T25780_001 and GSMUA_Achr9T30450_001 proteins have 15 sites (13Ser/2Thre and 9Ser/1Thre/5Tyr, respectively). Protein GSMUA_Achr2T14320_001 has 22 sites (10Ser/8Thre/4Tyr). Protein GSMUA_Achr9T23930_001 has 39 sites (23Ser/13Thre/3Tyr). Protein GSMUA_Achr5T03660_001 has the highest number of sites, 42 (21Ser/12Thre/9Tyr), which is expected for a phosphoribulokinase.

Protein phosphorylation is involved in several aspects of plant metabolism, including the response to abiotic and biotic stresses. An example is that, during the hypersensitive response (HR), there is protein phosphorylation, the generation of reactive oxygen species, and the production of phytoalexins, in addition to other essential chemical processes to provide an early defense response to a given disease [68,69].

Out of the characterized proteins, only GSMUA_Achr9T30450_001 did not denote N-type glycosylation sites, while the glycoprotein GSMUA_Achr9T23930_001 had the highest number of N-type glycosylation sites. The presence and localization of glycans in glycoproteins are important for the quality control of molecular folding (through chaperones), which directly influences the function of these polypeptides [70].

In addition, carbohydrate binding contributes to recognizing polypeptide chains by cell receptors, as they are configured as additional epitopes. Protein trafficking and host defense processes are among such recognition events, showing how important it is to detect these glycosylation sites in proteins involved in the defense response to diseases, including Black Sigatoka [71].

The number of amino acid residues (aa) of the proteins involved in the defense response to Black Sigatoka ranged from 132 (GSMUA_Achr9T30450_001) to 467 (GSMUA_Achr9T23930_001). The subcellular location identified for the GSMUA_Achr6T25780_001 and GSMUA_Achr9T30450_001 proteins was the chloroplast, as expected, since they are fundamental molecules in the photochemical phase of photosynthesis. The GSMUA_Achr2T14320_001 and GSMUA_Achr9T23930_001 proteins were detected in the cytoplasm due to their isoflavonoid biosynthesis role and glycolysis/gluconeogenesis pathways, respectively. The location of the GSMUA_Achr5T03660_001 protein was identified in plastids in general, among which the chloroplast itself is included. The mean hydropathicity (GRAVY), which refers to the water affinity of proteins, ranged from −0.336 to 0.014.

Unlike the proteins of the endoplasmic reticulum, plasma membrane, and the Golgi complex, the proteins present in plastids (chloroplasts and amyloplasts) and the cytoplasm are synthesized in free ribosomes of the cytosol, being the only ones devoid of a signal peptide. Thus, all the proteins characterized in this study did not present a signal peptide, a sequence capable of directing the protein to its subcellular location [72,73,74].

## 4. Conclusions

The results obtained herein show that the candidate genes involved in the defense response of bananas to Black Sigatoka, *psII* and *isr*, were upregulated during an incompatible interaction in resistant cultivars, namely, Calcutta-4 and Krasan Saichon.

Additionally, candidate genes *psI*, *psII*, and *isr* were downregulated earlier during a compatible interaction in the susceptible Grand Nain cultivar. However, the *psII* gene showed a late upregulation in this cultivar.

There was no significant expression of any of the genes involved in the defense response to Black Sigatoka in the susceptible cultivar Akondro Mainty.

Furthermore, the gene and protein sequences involved in the defense response to Black Sigatoka were analyzed, contributing to an understanding of its functions and mechanisms of action within the *Musa* spp. x *M. fijiensis* pathosystem.

The data discussed herein can support the Banana genetic breeding program in the development of breeding strategies, in addition to providing validated genes (*psII* and *isr*) involved in plant defense response, which may be used in marker-assisted selection and/or gene-editing strategies aiming to obtain banana cultivars more resistant to Black Sigatoka.

## Figures and Tables

**Table 1:** Banana cultivars used in this study and their characteristics.

Cultivar	Ploidy Level	Response to Black Sigatoka	References
Calcutta-4	AA	Resistant (R)	[16]
Grande Naine	AAA	Susceptible (S)	[17]
Krasan Saichon	AA	Resistant (R)	[6]
Akondro Mainty	AA	Susceptible (S)	[6]

**Table 2:** Primers of reference genes used for validation in banana genotypes.

Gene *	*Forward Primer* (5′-3′)	*Reverse Primer* (5′-3′)	*Amplicon* (bp)
*tubulin*	TGTTGCATCCTGGTACTGCT	GGCTTTCTTGCACTGGTACAC	112
25*S*	ACATTGTCAGGTGGGGAGTT	CCTTTTGTTCCACACGAGATT	106
*ef1*	CGGAGCGTGAAAGAGGAAT	ACCAGCTTCAAAACCACCAG	185

* *tubulin*: tubulin; *ef1*: elongation factor 1.

**Table 3:** Primers corresponding to the reference genes described by Podevin et al. [21].

Gene *	Name	*Forward*/*Reverse Primer* (5′-3′)	*Amplicon* (bp)
*psI*	GSMUA_Achr6G25780_001	F-TGCAGGAGCTCAATGACAAGA	80
		R-TGTGCCGAACTCCACTGTGT	
*psII*	GSMUA_Achr9G30450_001	F-CTTCTTGCTTGCAGCCCAAT	80
		R-CTTCCACCCTTACCTCCAACAT	
*isr*	GSMUA_Achr2G14320_001	F-CCTCCACGCCGAAAGATG	102
		R-GCAGGTCAATCCCTCTCAAC	
*utp*	GSMUA_Achr9G23930_001	F-GATCGCCAAGCTCCAATCC	93
		R-CAGCTCAGTTCCACGGACAA	
*prk*	GSMUA_Achr5G03660_001	F-CTTACGGCCCGGACACCTA	80
		R-AAGCAGCTGGCGATGGTAAC	
*act1*	GSMUA_Achr6G25350_001	F-TCTCAATCCCAAGGCAAACC	80
		R-TTGCTACATACATAGCCGGAACA	

* *psI*: subunit N of the center of the Photosystem I reaction; *psII*: Photosystem II; *isrl*: isoflavona reductase; *utp*: glycose-1-phosphate uridililtransferase; *prk*: phosphorribuloquinase; *act1*: actin 1.

**Table 4:** Introns and exons in the genes involved in the banana defense response to BLSD.

Number of Introns	Number of Exons	Genes
2	3	*psI*
4	5	*psII*
4	5	*isr*
20	21	*utp*
5	6	*prk*

**Table 5:** Open reading frame (ORF) of genes involved in banana defense response to BLSD.

Gene	ORF Position (bp)	Number of Nucleotides	Number of Aminoacids
*psI*	169:430	312	103
*psII*	379:525	147	48
*isr*	61:435	375	124
*utp*	7126:7449	324	107
*prk*	0.65625	885	294

**Table 6:** *Cis* elements responsive to biotic stress and their positions identified in the promotor region of genes involved in *Musa* spp. defense response to Black Sigatoka.

	*cis* Element (Position in Regard to The Start Codon)		
Gene	as-1	w Box	STRE
*psI*	1	1	3
	(195+)	(1015+)	(271−, 1436+, 841−)
*psII*	2	1	5
	(544+, 613−)	(1243+)	(236−, 583−, 499−, 354−, 528+)
*isr*	5	1	-
	(349−, 473+, 423+, 463+, 391+)	(508−)	
*utp*	4	1	-
	(41+, 1005+, 701+, 1405−)	(714−)	
*prk*	1	1	3
	(1033+)	(1141−)	(1198+, 1432−, 1346−)

**Table 7:** Characteristics of the proteins involved in defense response of bananas to Black Sigatoka.

Gene	Family	Protein *	Size of Protein (aa)	pI	Mw	Export Probability (%)	GRAVY *
*psI*	PSAN	GSMUA_Achr6T25780_001	173	9.13	18,415.84	Chloroplast	−0.336
						0.9148	
*psII*	PsbR	GSMUA_Achr9T30450_001	132	9.46	13,711.65	Chloroplast	−0.142
						0.9059	
*isr*	NmrA	GSMUA_Achr2T14320_001	312	6.11	34,034.04	Citoplasma	0.014
						0.6053	
*Utp*	UDPGP	GSMUA_Achr9T23930_001	467	5.57	51,346.02	Cytoplasm	−0.111
						0.8111	
*Prk*	PRK	GSMUA_Achr5T03660_001	442	8.27	49,598.94	Plastid	−0.32
						0.3952	

* The *Musa acuminata* v1 genome was used as reference.

## Data Availability

The data will be made available upon request.

## Supplementary Materials

The following supporting information can be downloaded at: https://www.mdpi.com/article/10.3390/cimb46120837/s1, Figure S1. (A) Standard curve for the 25S primer, Slope: −3.353. R2: 0.961, and 98.07 efficiency and (B) standard curve for the ACT1 primer, Slope: −3.613. R2: 0.914, and efficiency: 89.128%. Analysis generated by the 7500 (Applied Biosystems) software.
