# Supplementary material for: Expression of Genes Involved in Banana (Musa spp.) Response to Black Sigatoka
Source: Curr Issues Mol Biol. 2024 Dec 11;46(12):13991–4009. doi: 10.3390/cimb46120837 (PMC11726753; doi:10.3390/cimb46120837)
Supplement: Supplementary file 1 [file cimb-46-00837-s001.zip › Figure S1.pdf]

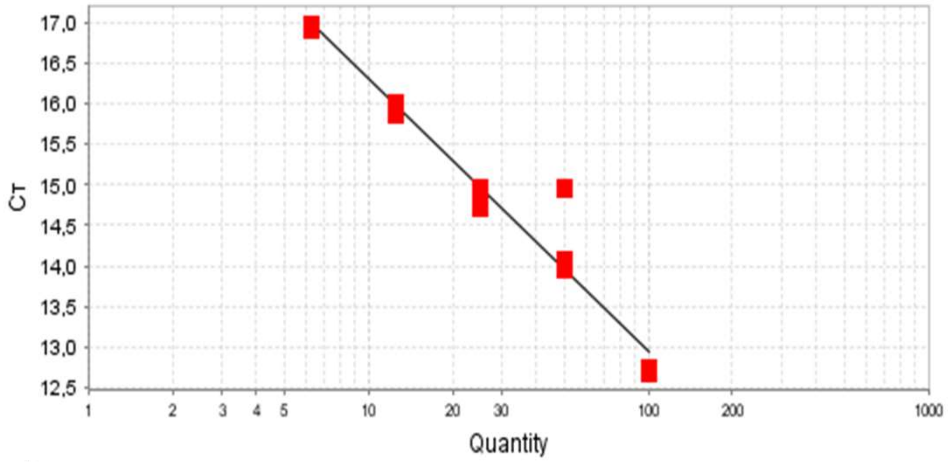

A)

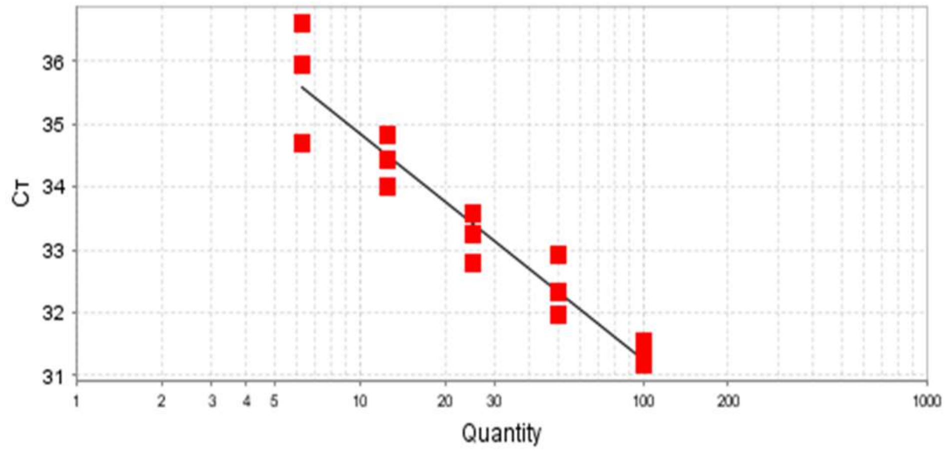

B)

Figure 1. A) Standard curve for the 25S primer, Slope: -3.353. R2: 0.961 and 98.07 efficiency and B) standard curve for the ACT1 primer, Slope: -3.613. R2: 0,914, efficiency: 89.128%. Analysis generated by the 7500 (Applied Biosystems) software.
